# Prevalence of polycystic ovary syndrome under NIH criteria among the tenth-grade Chinese schoolgirls in Guangzhou area: a cross-sectional epidemiological survey

**DOI:** 10.1186/s12905-023-02173-x

**Published:** 2023-01-21

**Authors:** Yu Hong, Ze-hong Zhou, Zhe Dong, Dong-zi Yang

**Affiliations:** 1 Department of Obstetrics and Gynecology, Memorial Hospital of Sun Yat-Sen University, Guangzhou, Guangdong People’s Republic of China; 2 Department of Obstetrics and Gynecology, Kiangwu Hospital, Kiangwu Road #33, Macau Special Administrative Region, People’s Republic of China

**Keywords:** Polycystic ovary syndrome, Adolescent, Prevalence, Epidemiology, Chinese

## Abstract

**Background:**

Polycystic ovary syndrome (PCOS) is currently considered to have a peri-adolescence onset and continuously influence the reproductive and metabolic health of the patients, while the diagnostic criteria among adolescent population haven’t been universally unified till now. This survey seeks to preliminarily evaluate the prevalence of PCOS in the tenth grade schoolgirls in Guangzhou area under NIH criteria and analyze the clinical features of adolescents with PCOS.

**Methods:**

The cross-sectional epidemiological survey was carried out among the tenth grade schoolgirls in Guangzhou area by the method of cluster sampling. The contents of this survey included the questionnaire, physical exams and serum measurements. Until now, totally 1294 girls underwent this survey and 1095 serum samples were restored. 235 non-hirsute (mFG < 6), postmenarcheally 2-year girls were randomly selected as the control group, among which the cut-off value of biochemical hyperandrogenemia was set accordingly. The prevalence of PCOS among this population was preliminarily evaluated according to the NIH criteria.

**Results:**

Along with the increase of gynecological age, the menstruations of girls was becoming more regular and the incidence of oligomenorrhea or amenorrhea was declining. Even among those who were less than 2 years after menarche, those whose menstrual cycle were longer than 90 days accounted for lower than 5%. The 95th percentile of mFG score was 6 among the girls who were < 2 years after menarche, and 5 among the girls who were > 2 years after menarche. Among the 235 healthy girls, the 95th percentile values of Testosterone (T), Free androgen index (FAI) and Androstenedione (A2) were 2.28 nmol/mL, 4.37, and 5.20 nmol/mL respectively. Based on the NIH criteria, the prevalence of PCOS in this survey was 3.86%. The prevalence of adolescent PCOS tend to slightly increase with age and gynecological age, but the difference was not statistically significant. The prevalence of PCOS among obese girls was markedly higher than that in lean girls.

**Conclusion:**

Based on the NIH criteria, the prevalence of PCOS among the tenth grade schoolgirls in Guangzhou area was 3.86%. The diagnosis of hyperandrogenism among adolescents should also be based on both clinical and biochemical parameters.

## Background

Polycystic ovarian syndrome (PCOS) is the most common endocrinopathy, defined by the presence of chronic anovulation and hyperandrogenism after the exclusion of secondary causes, with an estimated prevalence of approximately 6% in premenopausal women [1]. In addition to reproductive and hyperandrogenic concerns, PCOS is also associated with a broad range of long-term adverse sequalae, including insulin resistance (IR), dyslipidemia, glucose intolerance and hypertension [2]. It was once thought to affect the appearance and fertility primarily of the adult women, but is currently recognized to have a peri-adolescence onset and continuously influence the patient’s whole life [3].

Since its original description in 1935, the definition of PCOS has undergone three major revisions, including the National Institues of Health (NIH) consensus conference held in 1990 [4], the Rotterdam European Society for ESHRE/ASRM- sponsored PCOS consensus workshop group held in 2003 [5], and the Androgen Excess and PCOS Society in 2006 [6]. These three criteria have established similar diagnostic points based on the specific pathophysiological changes of PCOS, but with different emphasis. In addition, none of the above groups has put forward the diagnostic criteria of PCOS specifically for adolescent population, probably due to the overlapping between pathophysiological feature in PCOS adolescents and normal pubertal physiology. The application of PCOS criteria for adult women among adolescent population would be probably not enough to reflect the speciality of adolescent PCOS. Some experts have ever raised their own opinions on the diagnosis of adolescent PCOS [7, 8], which were generally stricter than adult standard to avoid inappropriate premature diagnosis, while none has obtained the consent from any expert panel or society in this area. Accordingly, the absence of unified criteria leads to the scarcity of literature and reports over the epidemiological data either domestically or abroad.

Considering the various metabolic abnormalities related to PCOS and the early onset of PCOS during peri-adolescence, it is quite meaningful to make early diagnosis and take early measures on PCOS during puberty in order to guarantee the reproductive and physical health in the later life. Most of the reports on adolescent PCOS result from the patients in hospitals, while the materials from the general population are quite few. Therefore, according to the cross-sectional epidemiological survey design, we carried out a large-scale, cluster sampling study in Guangzhou area to explore the incidence and related clinical characteristics of PCOS in adolescent girls, and to collect experiences on clinical diagnosis of adolescent PCOS.

## Methods

### Subjects

#### Survey subjects

Base on Consensus on women's health aspects of PCOS: the Amsterdam ESHRE/ASRM-Sponsored 3rd PCOS Consensus Workshop Group, the confirmation of PCOS should be considered only in girls who had menarche at least 2 years before diagnosis [9]. Therefore, the target population of this study was set as the tenth-grade girls by cluster sampling, ie this study enrolled all the tenth-grade schoolgirls from 5 middle schools randomly assigned in five different districts of Guangzhou. In cooperation with Guangzhou Primary and Secondary School Health Care Center, the research team dispatched specially trained gynecologists to the school site or in the health care center to conduct investigations with the medical examination team.

#### Sample size calculation

The sample size was calculated using the formula

$${\text{N}}\, = \,u^{2} p\left( {{1} - p} \right)/\delta^{2} ,$$

in which P = prevalence 2.2%, taken from our previous study [10]. *δ* = margin of error, 1%. *u* = 1.96 at 95% confidence interval. Accordingly N = required sample size = 1.96^2^ × 0.022x (1 − 0.022)/0.01^2^ = 827. Considering a non-response rate of 20%, the sample size was 1034.

### Survey protocol

#### Questionnaire survey

An epidemic questionnaire on the incidence of adolescent PCOS was applied in this study, and was appropriately simplified on the basis of the epidemic questionnaire on the incidence of adult PCOS in our previous research [10], The design of the original questionnaire is the result of extensive discussion among statisticians, epidemiologists and multi center clinical medical experts, which meets the requirements of relevant data collection, data analysis, epidemiological investigation standards and clinical diagnosis.

The current questionnaire includes the following parts: (1) Basic personal information; (2) General conditions: including menstrual conditions (menarche age, appearance of regular menstrual after menarche, menstrual cycle days, menstrual period, menstrual volume, dysmenorrhea, etc.), previous living habits (eating habits, exercise habits), previous medical history, family conditions (diabetes, hypertension, menstrual conditions of mother and sister, early baldness of father and brother).

#### Physical examination

All participants underwent anthropometric parameters measurements. The average of two measurements of blood pressure (BP) with the subject in the sitting position was taken at 5 min intervals after resting for at least 15 min. Height and weight were measured in light clothing without shoes by a digital electronic scale. Body mass index (BMI) was calculated as weight in kilograms divided by the square of height in meters. Waist and hip circumferences were measured at the narrowest and widest parts at the level of umbilicus and buttocks respectively, and then waist-hip ratio (WHR) was calculated. The presence of hirsutism was scored in every girl by a same investigator, using a modification of the Ferriman-Gallwey method, according to a given diagram [11]. Thyroid and breast examination were done by palpation.

#### Hormonal measurements

Plasma free-testosterone (FT), sex hormone binding globulin (SHBG), Androstenedione (A2), and 17-hydroxyprogesterone (17-OHP) were measured by enzyme-linked immunosorbent assay (ELISA). Testosterone (T), prolactin (PRL) and thyroid stimulating hormone (TSH) were measured by chemiluminescence. Free androgen index (FAI) was calculated using the formula [serum testosterone (nmol/L) × 100/SHBG (nmol/L)] [12].

### Definition criteria of PCOS

Due to no unified criteria for adolescent PCOS around the world, the presence of PCOS was defined in this study by: (1) oligo/anovulation(≤ 8 cycles per year, or menstrual cycle < 21 days or > 35 days in length, without taking hormonal contraception); (2) evidence of clinical hyperandrogenism (hirsutism (F-G Score ≥ 6), or acne or androgenic alopecia) and/or hyperandrogenemia (serum TT, A2 and FAI levels higher than the 95th percentile of the girls randomly selected in this study presenting no clinical evidence of hyperandrogenism or menstrual disturbances); and (3) for those girls suspected of PCOS, serum TSH, PRL, 17-OHP were measured subsequently for ruling out thyroid diseases (TSH outside the reference range (0.35–5.5 mIU/L)), hyperprolactinemia (PRL > 24 μg/L), and congenital adrenal hyperplasia (17-OHP > 6 nmol/L).

### Statistical analysis

Epidata3.02 software was applied to build the database of the questionnaire obtained from the survey, arranging and entering data, and then checking with reference to the original questionnaire to ensure the correctness of data entry. SPSS13.0 analysis software was used for statistical processing of data. The Kolmogorov-Smirnoff normality test was used to test the distribution of continuous variables. If it conformed to the normal distribution, it was statistically described with the mean ± standard deviation. If it was a skew distribution, it was expressed as the median (upper quartile ~ lower quartile). Chi square was used to calculate the rate. Pearson chi square was used to compare the rates between the two groups; Kruskal Wallis rank sum test was used to compare the rates among multiple groups, and Bonferroni method was used to compare the rates. Two-tailed *P* < 0.05 was considered statistically significant.

## Results

**Fig. 1:**
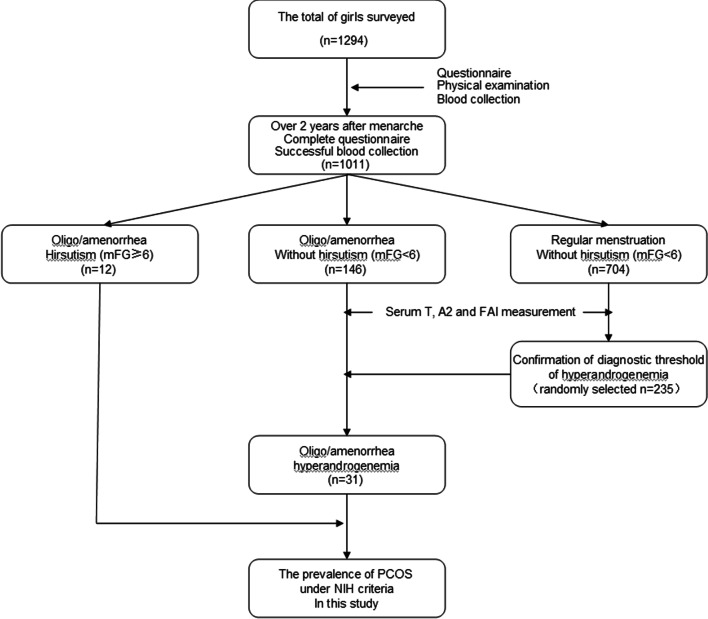
Flowchart of this survey

From January 2012 to April 2014, a total of 1294 tenth-grade schoolgirls in 5 senior high schools in Baiyun District, Haizhu District, Tianhe District, Liwan District and Yuexiu District of Guangzhou were investigated, in which 199 girls had a physical examination in the afternoon and were not fasting, so 1095 serum samples were kept for further tests. Their age ranged between 14 and 18 years with a mean age of 15.8 years, and mean body mass index was 20.72 ± 2.56 kg/m^2^. Information about previous living habits, previous medical history and family conditions in this adolescent population was found to be similar to that in the adult counterpart from the previous survey we performed [10]. Figure 1 outlined the survey procedure and outcomes.

### Incidence of oligomenorrhea or amenorrhea (categorized by years after menarche)

As shown in Table 1, after categorizing the surveyed girls according to the number of years after menarche, with the increase of the number of years after menarche, the proportion of girls with regular menstruation is increasing, the proportion of oligomenorrhea is descending, and the proportion of regular menstruation after 3 years of menarche is significantly higher than that before 2 years of menarche; Moreover, the incidence of oligomenorrhea was significantly lower in those who were over 2 years after menarche than in those who were within 2 years after menarche; Even among the girls within 2 years after menarche, the proportion of menstrual cycle more than 90 days is no more than 5%.

**Table 1:** Current menstrual condition of surveyed schoolgirls (categorized by years after menarche)

	< 2 years after menarche (n = 84)	3rd year after menarche (n = 275)	> 3 years after menarche (n = 736)
Regular menstruation [n(%)]	58 (69.0)	218 (79.3)	606 (82.3)^b^
Irregular menstruation [n(%)]	26 (31.0)	57 (20.7)	131 (17.8)^b^
Oligo/amenorrhea [n(%)]	24 (28.6)	49 (17.8)^a^	109 (14.8)^b^
Cycle length > 90 days [n(%)]	4 (4.8)	3 (1.1)	2 (0.3)^b^

^a,b^When compared with the group within 2 years after menarche

### Clinical/biochemical hyperandrogenism

#### Clinical hyperandrogenism

The diagnostic criteria of clinical hyperandrogenism had not been unified universally, and hirsutism is still considered as the main index. Most studies regard hirsutism and acne as the manifestations of clinical hyperandrogenism if either of them was identified. In this study, the 95th percentile of mFG score of girls within 2 years after menarche is 6, while the 95th percentile of mFG score of girls both in the third year and over 3 years after menarche is 5. Therefore, this study took mFG 6 as the diagnostic threshold, and mFG ≥ 6 as hirsutism, i.e., clinical hyperandrogenism. Details is shown in Table 2.

**Table 2:** 95th percentile of mFG score (categorized by years after menarche)

	< 2 years after menarche (n = 84)	3rd year after menarche (n = 275)	> 3 years after menarche (n = 736)
mFG point (95th percentile)	6	5	5

#### Biochemical hyperandrogenism (hyperandrogenemia)

At present, there is no unified index to define biochemical hyperandrogenism around the world. The indexes used in the literature are different, including T(Testosterone), FT(Free testosterone), A2(Androstenedione), DHEAS(Dehydroepiandrosterone sulfate) and FAI(Free androgen index). Recent studies suggested that there were obvious defects in the FT test method, the comparability of test results between various laboratories was not high [13], and the diagnostic value of DHEAS for hyperandrogenemia in hirsute women is not high [14]. Therefore, the androgen indexes detected in this study are T, A2 and FAI, and if either of them elevated beyond the normal range, hyperandrogenemia was diagnosed. Among the 1294 girls surveyed, 704 had regular menstruation and no hirsutism for two years after menarche. The serum of 235 girls (set as normal control subgroup) were randomly selected for the detection of androgen indexes. According to the test of normal distribution, T, A2 and FAI in these 235 normal girls are non normal distribution. Therefore, the 95th percentile of androgen level of these girls is taken as the cutting point, i.e., ≥ the 95th percentile is hyperandrogenemia, as shown in Table 3.

**Table 3:** Percentile of T, A2, FAI in normal control girls

Percentile	T (nmol/L)	A2 (nmol/L)	FAI
25	0.89	1.48	1.28
50	1.23	2.27	1.91
75	1.61	3.27	2.71
90	2.05	4.69	3.70
95	2.28	5.20	4.37

#### The prevalence of PCOS in this study

We finally recruited 1011 schoolgirls who were in over 2 years after menarche, with 158 of them being oligo/amenorrhea. Further, the above biochemical hyperandrogenism indexes (T, A2, FAI) and clinical hyperandrogenism index (mFG ≥ 6) were combined to calculate the prevalence of PCOS according to NIH standard under different combinations. See Table 4 below for details.

**Table 4:** Summary of PCOS prevalence in different hyperandrogenism index combinations

Definition of hyperandrogenism	Number of adolescent PCOS (n)	NIH-PCOS prevalence % (n/N)
Hyperandrogenism 1^a^	21	2.08 (21/1011)
Hyperandrogenism 2^b^	29	2.87 (29/1011)
Hyperandrogenism 3^c^	39	3.86 (39/1011)

^a^Either of T and mFG elevated

^b^Any of T, FAI and mFG elevated

^c^Any of T, FAI, A2 and mFG elevated

It could be seen from the above statistical results that the calculated prevalence of PCOS was slightly different with various indexes of biochemical and/or clinical hyperandrogenism. The overall trend was that the more indexes included, the more individuals detected as hyperandrogenism, so that according to the NIH criteria, the prevalence of PCOS increased from 2.08 to 3.86%. Considering the fact that T, A2 and FAI had been detected and the mFG score diagram was applied in this study, hyperandrogenism 3 was identified as the index to diagnose biochemical and/or clinical hyperandrogenism. Therefore, the incidence rate of PCOS under the NIH criteria was 3.86% (95CI 3.18–4.54%).

#### The prevalence of PCOS categorized by age and gynecologic age

As shown in Table 5, although there is no significant difference in the prevalence of PCOS among different ages, on the whole, the prevalence shows a slight increase trend with the increase of age (due to the small amount of girls aged 14 and 18, they are not included in the table), *P* > 0.05.

**Table 5:** Comparison of NIH-PCOS prevalence between different ages

	15-year-old	16-year-old	17-year-old
NIH-PCOS girls (n)	13	22	3
Non-PCOS girls (n)	331	555	74
NIH-PCOS prevalence (%)	3.78	3.81	3.9

As shown in Table 6, although there is no significant difference in the prevalence of PCOS among different gynecologic ages [15], on the whole, the prevalence shows a slight increase trend with the increase of gynecologic age (due to the small amount of girls who were over 5 years after menarche, they are not included in the table), *P* > 0.05.

**Table 6:** Comparison of NIH-PCOS prevalence between different gynecologic ages

	3rd year	4th year	5th year	6th year
NIH-PCOS girls (n)	7	15	12	4
Non-PCOS girls (n)	268	357	267	66
NIH-PCOS prevalence (%)	2.55	4.03	4.30	5.71

#### The prevalence of PCOS categorized by BMI

When BMI ≥ 23.0 kg/m^2^ was regarded as overweight and BMI ≥ 25.0 kg/m^2^ was regarded as obesity diagnostic criteria, 29 of 915 normal weight girls were diagnosed with PCOS, with a prevalence rate of 3.2%, while 7.3% and 14.6% respectively in overweight and obese girls. Therein, the prevalence of PCOS in obese girls was significantly higher than that in normal weight girls (*P* < 0.05). Details is shown in Table 7.

**Table 7:** The prevalence of NIH-PCOS categorized by BMI

	Normal weight girls (BMI < 23.0 kg/m^2^)	Overweight girls (23.0 ≤ BMI < 25.0 kg/m^2^)	Obese girls (BMI ≥ 25.0 kg/m^2^)
NIH-PCOS girls (n)	29	4	6
Non-PCOS girls (n)	886	51	35
NIH-PCOS prevalence (%)	3.2	7.3	14.6^a^

^a^When compared with normal weight girls, *p* < 0.05

## Discussions

At present, the data about the incidence rate of PCOS throughout the world had been obtained intensively from reproductive-aged female population. Due to the differences in diagnostic criteria, sampling methods and the heterogeneity of PCOS itself, the prevalence rates, retrieved by domestic and foreign researchers in the epidemiological investigations on PCOS, vary greatly. The prevalence rate of PCOS among women of reproductive age in various countries was reported to be between 5 and 13% [16]. In a newly published meta-analysis in Chinese women, Wu et al. analyzed 69 studies with totally 154,599 participants enrolled from the regional population survey to understand the prevalence of PCOS in different regions and different population fully. In result, the overall prevalence of PCOS was 10.01% (95% CI 8.31–11.89%), and it varied with different regions, occupation/identity, age, time of publication, diagnostic criteria, survey time, which is coincidence with the current knowledge of PCOS [17].

Until now, large-sample epidemiological study on puberty PCOS was quite limited, which could be due to the absence of universally unified diagnostic criteria for adolescent PCOS. Nidhi et al. [18] prospectively investigated 460 girls aged 15–18 years from a residential college in India. By taking T > 2.85 nmol/L as the standard for biochemical hyperandrogenism, mFG ≥ 6 as the standard for clinical hyperandrogenism, and presence of > 10 follicles, 2–8 mm in diameter, or ovarian volume > 10m^3^ as the standard for ultrasonic PCO, the survey results showed that the incidence of adolescent PCOS was 9.13% under Rotterdam criteria and 2.61% under NIH criteria. Recently, Shama et al. [19] conducted systematic review and meta-analysis to estimate the pooled prevalence of PCOS among Indian girls aged 14–19 years, including 12 studies and totally 4473 participants. Approximately one in five Indian adolescent girls were diagnosed using NIH criteria, AES criteria or Rotterdam criteria. In a sub-study of comprehensive health and lifestyle studies of young Australian women (aged 16–29 years), Varanasi et al. [20] recruited the participants via Facebook advertising to complete questionnaires, physical examinations and blood tests. Therein, mFG ≥ 8 and T > 2.2 nmol/L were defined as clinical and biochemical hyperandrogenism respectively. According to NIH criteria, the prevalence of PCOS was 12% (31/254) in this study and 14.3% (7/63) in aged 16–20 subgroup. In Chinese scholar Wu’s meta-analysis report, with statistics from 1995 to 2020, the pooled prevalence rate of PCOS was 10.26% and 3.01% in individuals aged 10–20, and in school-based studies, respectively. Hence, diagnosing PCOS in adolescents is more challenging, because anovulatory cyclic bleeding and multi-follicular ovaries are common during puberty and considered as part of normal pubertal physiology, while the application of adult criteria results in a high prevalence and may lead to over-diagnosis and unnecessary anxiety among adolescents. According to the international evidence-based guideline for the assessment and management of PCOS in 2018, both oligo-anovulation and hyperandrogenism are required, with ultrasound not recommended for diagnosis of PCOS in adolescents, i.e., NIH criteria [21].

Until now, the definition of biochemical hyperandrogensim has not been universally unified yet. None of the well-konwn guidelines did specify which androgen indicator should be tested and which detection method should be used. Total testosterone was the most commonly used parameter. However, the sensitivity of the measuring method is not high, and the normal range and normal value corresponding to age are lacking, making it difficult to compare the data measured by different laboratories.FAI is recently considered as a more sensitive evaluation method, which can distinguish PCOS patients from healthy people. Hence, in this study, we took mFG ≥ 6 as the definition for clinical hyperandrogenism, and T > 2.28 nmol/L as one of the criteria for biochemical hypersandrogenism. At the same time, A2 and FAI were also used as reference indexes for diagnosing biochemical hyperandrogenism. According to NIH criteria, the prevalence of PCOS among the tenth-grade schoolgirls in Guangzhou is 3.86%, which is slightly higher than the result from Indian scholars under the similar research background (Asian population, NIH criteria, school-based). The slight discrepancy might be caused by that we tested three androgen indexes: T, A2 and FAI, which expanded the scope of androgen measurement, leading to detect more hyperandrogenemia individuals.

What’s more, we also noted that the incidence rate of PCOS in this survey increased slightly with age and gynecologic age, which was worthy of our vigilance. According to our study: (1) with the increase of age and gynecologic age, the menstrual cycle of adolescent girls gradually becomes regular, and the incidence of oligomenorrhea or amenorrhea is decreasing; (2) with the increase of age and gynecologic age, the incidence of hirsutism remained quite stable; (3) with the increase of gynecologic age, the serum androgen indexes increased significantly. The above results suggest that the transcending tendency of the incidence of adolescent PCOS with age and gynecologic age could be mainly due to the increase in the incidence of biochemical hyperandrogenism. Therefore, some scholars proposed that it could be better to diagnose adolescent PCOS according to hyperandrogenemia rather than the clinical manifestations of hyperandrogensim [8]. We also agreed with this view. However, due to the lack of the normal range of androgen indicators in adolescent population, researchers should formulate their own reference values according to the local population, otherwise the definition of adolescent biochemical hyperandrogenism would be arbitrary.

The present study also had certain limitations. Firstly, since the vast majority of girls in middle school were minors and under the double guardianship of the school and their parents, the actual implementation process was limited to questionnaires, physical examinations and serum collection for objective reasons, and no trans-abdominally or trans-rectally pelvic ultrasonography examination was performed. Secondly, considering that this study was a cross-sectional study without random sampling, and the age of the surveyed girls was quite limited, the incidence of adolescent PCOS in Guangzhou area could not be accurately obtained, so it is necessary to make further efforts to carry out standardized epidemiological investigation and research in the future.

## Conclusions

This large scale epidemiological survey reveals that, based on the NIH criteria, the prevalence of PCOS among the tenth grade schoolgirls in Guangzhou area was 3.86%. Although it seems lower than that in other ethnic groups and regions, it is worthwhile to pay more attention on this specific age population. Meanwhile, the diagnosis of hyperandrogenism among adolescents should also be based on both clinical and biochemical parameters.

## Data Availability

The datasets generated during and/or analyzed during the current study are available from the corresponding author on reasonable request.

## References

[1] Ding T, Hardiman PJ, Petersen I, Wang FF, Qu F, Baio G (2017). The prevalence of polycystic ovary syndrome in reproductive-aged women of different ethnicity: a systematic review and meta-analysis. Oncotarget.

[2] Rajkumari P, Sahoo J, Sujata P, Sahoo G, Hansa J (2016). Awareness about PCOS and the likelihood of its symptoms in adolescent girls in a semi-urban set-up: a cross sectional study. J Med Sci Clin Res.

[3] Balen AH, Conmwy GS, Homburg R (2005). Polycystiec ovary syndrome.

[4] Zawadski JK, Dunaif A (1992). Diagnostic criteria for polycystic ovary syndrome: towards a rational approach.

[5] Rotterdam ESHRE/criteria and long-term health ASRM-Sponsored PCOS Consensus Workshop Group. Revised 2003 consensus on diagnostic criteria and long-term health risks related to polycystic ovary syndrome FertilSteril. 2004;81:19–25. 10.1016/j.fertnstert.2003.10.004 14711538

[6] Azziz R, Carmina E, Dewailly D, Diamanti-Kandarakis E, Escobar-Morreale HF, Futterweit W, Janssen OE, Legro RS, Norman RJ, Taylor AE, Witchel SF; Task force on the phenotype of the polycystic ovary syndrome of the androgen excess and PCOS society. The androgen excess and PCOS Society criteria for the polycystic ovary syndrome: the complete task force report. Fertil Steril. 2009; 91(2):456–88. 10.1016/j.fertnstert.2008.06.035. 10.1016/j.fertnstert.2008.06.035 18950759

[7] Sultan C, Paris F (2006). Clinical expression of polycystic ovary syndrome in adolescent girls. Fertil Steril.

[8] Carmina E, Oberfield SE, Lobo RA (2010). The diagnosis of polycystic ovary syndrome in adolescents. Am J Obstet Gynecol.

[9] Fauser BC, Tarlatzis BC, Rebar RW, Legro RS, Balen AH, Lobo R, Carmina E, Chang J, Yildiz BO, Laven JS, Boivin J, Petraglia F, Wijeyeratne CN, Norman RJ, Dunaif A, Franks S, Wild RA, Dumesic D, Barnhart K (2012). Consensus on women's health aspects of polycystic ovary syndrome (PCOS): the Amsterdam ESHRE/ASRM-sponsored 3rd PCOS consensus workshop group. Fertil Steril.

[10] Chen X, Yang D, Mo Y, Li L, Chen Y, Huang Y (2008). Prevalence of polycystic ovary syndrome in unselected women from southern China. Eur J Obstet Gynecol Reprod Biol.

[11] Hatch R, RosenWeld RL, Kim MH, Tredway D (1981). Hirsutism: implications, etiology, and management. Am J Obstet Gynecol.

[12] Mathur RS, Moody LO, Landgrebbe S, Williamson HO (1981). Plasma androgens and sex hormone binding globulin in the evaluation of hirsute patients. Fertil Steril.

[13] Rosner W, Auchus RJ, Azziz R, Sluss PM, Raff H (2007). Position statement: utility, limitations, and pitfalls in measuring testosterone: an Endocrine Society position statement. J Clin Endocrinol Metab.

[14] Willenberg HS, Bahlo M, Schott M, Wertenbruch T, Feldkamp J, Scherbaum WA (2008). Helpful diagnostic markers of steroidogenesis for defining hyperandrogenemia in hirsute women. Steroids.

[15] Kaplanoglu M, Bülbül M, Konca C, Kaplanoglu D, Tabak MS, Ata B (2015). Gynecologic age is an important risk factor for obstetric and perinatal outcomes in adolescent pregnancies. Women Birth.

[16] Bozdag G, Mumusoglu S, Zengin D, Karabulut E, Yildiz BO (2016). The prevalence and phenotypic features of polycystic ovary syndrome: a systematic review and meta-analysis. Hum Reprod.

[17] Wu Q, Gao J, Bai D, Yang Z, Liao Q (2021). The prevalence of polycystic ovarian syndrome in Chinese women: a meta-analysis. Ann Palliat Med.

[18] Nidhi R, Padmalatha V, Nagarathna R, Amritanshu R (2011). Prevalence of polycystic ovarian syndrome in Indian adolescents. J Pediatr Adolesc Gynecol.

[19] Sharma M, Khapre M, Saxena V, Kaushal P (2021). Polycystic ovary syndrome among Indian adolescent girls: a systematic review and metanalysis. Nepal J Epidemiol..

[20] Varanasi LC, Subasinghe A, Jayasinghe YL, Callegari ET, Garland SM, Gorelik A, Wark JD (2018). Polycystic ovarian syndrome: prevalence and impact on the wellbeing of Australian women aged 16–29 years. Aust N Z J Obstet Gynaecol.

[21] Teede HJ, Misso ML, Costello MF, Dokras A, Laven J, Moran L, Piltonen T, Norman RJ; International PCOS Network. Recommendations from the international evidence-based guideline for the assessment and management of polycystic ovary syndrome. Fertil Steril. 2018;110(3):364–379. 10.1016/j.fertnstert.2018.05.004 10.1016/j.fertnstert.2018.05.004 PMC6939856 30033227

